# Interactions between multi-species marine predator aggregations and the energy landscape

**DOI:** 10.1098/rstb.2025.0195

**Published:** 2026-10-15

**Authors:** Yannis P. Papastamatiou, Johann Mourier, Gil Iosilevskii

**Affiliations:** ^1^ Institute of Environment, Department of Biological Sciences, Florida International University, North Miami, FL, USA; ^2^ MARBEC, Univ Montpellier, CNRS, IFREMER, IRD, Sète, France; ^3^ Faculty of Aerospace Engineering, Technion, Haifa, Israel

**Keywords:** sharks, foraging, upwelling, assortativity, marginal value theory, optimal foraging theory

## Abstract

Multi-species predator aggregations can be found in both coastal and pelagic ecosystems, forming for both foraging and refuging (resting) purposes. The energy landscape represents the power costs required to move through or reside within a habitat. As species may differ in their morphology, buoyancy and length, there may be species-specific heterogeneity in their energy landscapes. Here, we highlight how the energy landscape may influence the small-scale distribution of predators within an aggregation and potentially their ability to forage. We focus on negatively buoyant predators, such as sharks, which can reduce their metabolic costs by swimming in upwelling currents. We develop a biomechanical model to show how shark length and buoyancy alter the energetic benefit sharks obtain from swimming in upwelling currents. We provide evidence of small-scale spatial separation between shark species within a French Polynesian channel, which may be related to species exploiting optimal energetic environments. Second, we show how the energy landscape influences patch residence times in foraging predators and how this may differ between species. For instance, different species within a multi-species foraging aggregation may quit the patch at different prey densities. Overall, the energy landscape may help to drive the distribution of species within aggregations or even inter-species patterns of assortativity.

This article is part of the theme issue ‘Multi-predator feeding aggregations in pelagic waters’.

## 1. Introduction

An animal's ‘energy landscape’ consists of a spatial representation of its power requirements to move through and reside within habitats [1]. More specifically, the energy landscape reflects spatial changes in the cost of transport—the energy required to move a unit distance—which can be defined by the animal's metabolic rate/movement speed. Optimal foraging theory predicts that animals will forage to maximize their energy surplus (subject to constraints) and will select habitats that minimize their metabolic costs (all other things being equal) when travelling or refuging. Consequently, the energy landscape has been used to explain animal movement paths, habitat selection and foraging ranges in both terrestrial and marine ecosystems [1–3]. Within the marine environment, energy landscapes include factors such as depth, current strength, flow dynamics, travel distances, seafloor topography, oxygen concentration and water temperature [3–7]. There will be strong spatial heterogeneity in the energy landscape of coastal environments; for example, water current dynamics and temperature vary with depth and also bottom topography (e.g. upwelling sites) [8]. Energy landscapes can also be temporally dynamic, as current strength and direction may change rapidly, several times per day with changing tides [6,8,9].

Multi-species aggregations of marine predators are common and may be related to foraging and/or refuging (resting) purposes (e.g. [10–14]). Interactions within these aggregations may be competitive, kleptoparasitic, predator–prey or mutualistic [11,15,16]. Multi-species aggregations may include species with dramatic differences in body size, shape and buoyancy, which all influence their energetics [17]. As such, the energy landscape for species within the aggregations may differ substantially depending on their swimming mechanics, metabolic costs and thermal physiology. The role of physiological traits (e.g. aerobic scope, lateralization) in patterns of assortment in fish shoals has already been considered [18], but less is known about how morphological and physiological traits interact with their environment and contribute to assortment patterns. The ability of multi-species aggregations to form, the spatial distribution of species within the aggregation, and even patterns of assortativity may strongly depend on their energy landscape. If the multi-species aggregation is for foraging, then behaviours such as patch residence times will also depend on the energy landscape, via the energetic cost of foraging [7,19].

Here, we highlight two mechanisms by which the energy landscape may interact with multi-species predator aggregations, under both a refuging and foraging context. We identify potential mechanisms by which the energy landscape may influence the distribution of predators within aggregations or their patch residence times while foraging.

## 2. Refuging in predators that never stop swimming

Sharks may form multi-species aggregations, some of which do not appear to be foraging-related (e.g. [13]). Most sharks are negatively buoyant and rely on hydrodynamic lift generated over stiff surfaces, such as pectoral fins, to offset gravity. Many species of reef and pelagic sharks are obligate ram ventilators and must swim continuously to obtain sufficient oxygen uptake rates. Tidal upwelling currents, where water is deflected from the seafloor and moved upwards, may reduce the swimming effort in negatively buoyant animals in the same way that updrafts reduce the flying effort of soaring birds [6,20]. Upwelling sites may therefore function as refuging areas: non-foraging habitats where the animals minimize their activity and metabolic costs [6]. While several shark species are morphologically similar (e.g. Carcharhinid sharks), they may still differ significantly in terms of their length and buoyancy [17]. Under these scenarios, the energetic cost of swimming in an upwelling current will depend on how these parameters differ between individuals and species. We develop a modelling framework to predict how shark morphology will determine the energetic advantage that individuals obtain from swimming in currents or upwelling. Our framework is an extension of the one developed for soaring and gliding birds [20].

### (a) The model

Hydrodynamic forces acting on a swimming shark are typically divided into lift (*L*) and drag (*D*) components, the former acting perpendicular to the direction in which the shark swims and the latter parallel to it (figure 1). Both components can be expressed using the respective hydrodynamic coefficients, *C_L_* and *C_D_*, with

(2.1) $$L=\left(1/2\right)\rho v^{2}SC_{L}$$

and

(2.2) $$D=(1/2)\rho v^{2}SC_{D},$$

in which *S* is a reference area (it can be any area, but we used the maximal cross section of the body as used in other shark studies [17,21,22], which is convenient when used in conjunction with the prismatic coefficient introduced below), *ρ* is the density of water and *v* is the swim speed. Both coefficients are functions of the angle of attack, which is set by the shark adjusting its body posture and the orientation of its fins relative to the body (figure 1a,b).

**Figure 1.**
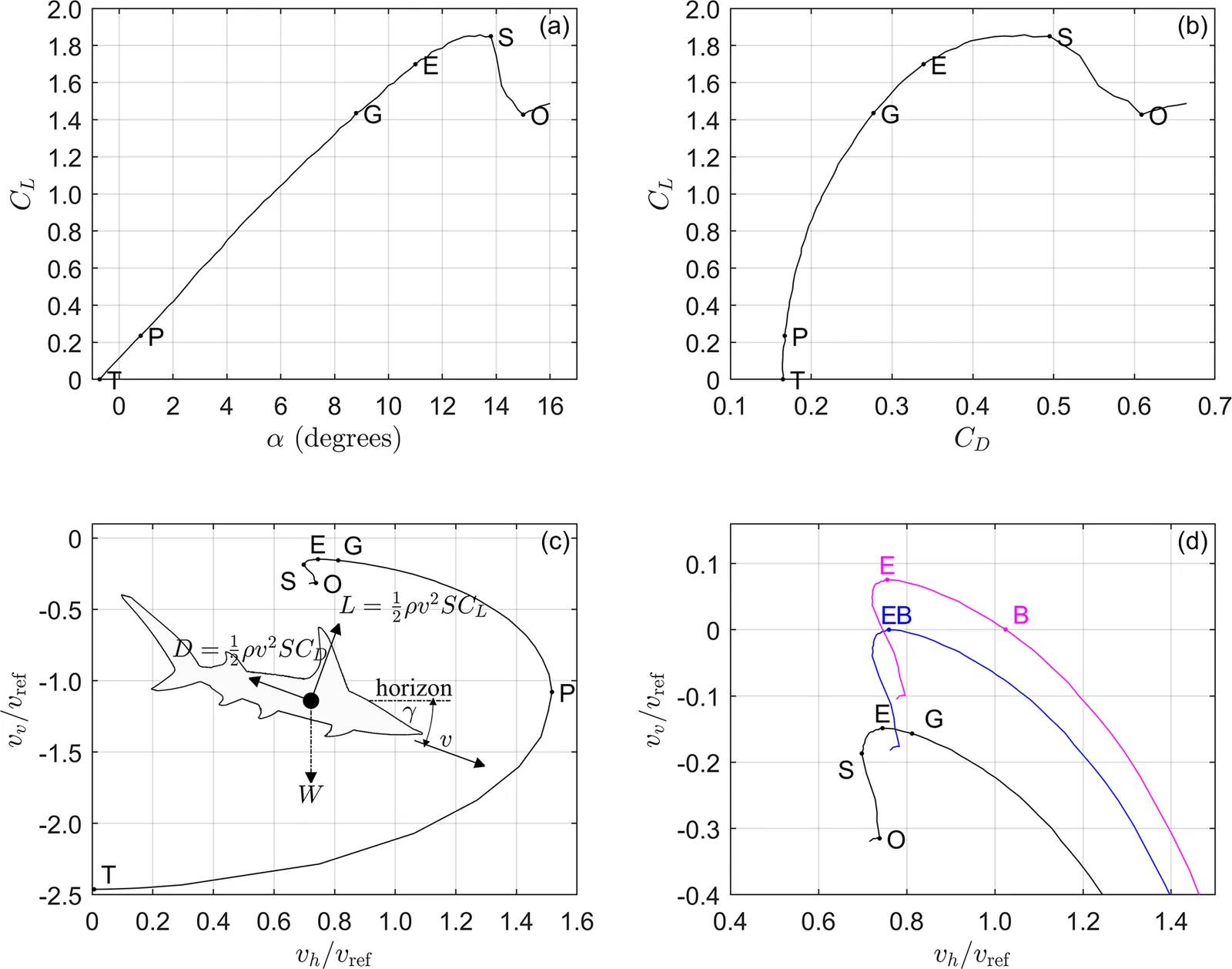
(a) Lift coefficient (C_L_) of a model Carcharhinid shark as a function of the angle of attack (*α*), (b) drag coefficient (C_D_) of the same shark as a function of the lift coefficient. Both coefficients are determined in relation to the maximum cross-sectional area of the body. The data are in the supplementary material, figure S1 of publication [21]. A shark sets its angle of attack by adjusting its body posture and the angles of its fins relative to the body. For each angle of attack, the combination of lift and drag coefficients sets the speed of the shark in a steady glide, *v*, and its angle *γ* relative to the horizon. The respective velocity components (horizontal *v_h* on the *x* axis, vertical *v_v* on the *y* axis. *v_ref* is the reference speed) are plotted against each other in (c), forming what is known as a ‘snail’ diagram defined in equation (2.7). In a steady glide, a shark can balance itself at any combination of *v_h* and *v_v* found on the curve, but not elsewhere. Points mark a post-stall (O), the verge of a stall (S), the minimal sink rate (E), the best glide angle (G), the maximal penetration (horizontal) speed (P) and the terminal speed (T), respectively. The black line on plate (d) is a magnified version of (c). To stay on the coloured curves in (d), a shark needs to add power. The minimal power that allows it to swim at constant depth corresponds to the same swim speed at which its sink rate was minimal when gliding (point E on the black curve), marked by EB on the blue curve. Adding more power enables the shark to ascend (e.g. point E on the magenta curve) or to swim faster at a constant depth (point B on the magenta curve).

Generating hydrodynamic forces obviously requires moving relative to water. Movement requires power, and if this is not provided by active swimming, a shark will descend through the water column in the same way that a soaring bird will descend in still air. The (stabilized) swim speed of a shark *v* and its swim path angle relative to the horizon *γ* (see the inset to figure 1c) are solutions of the pair of equilibrium conditions:

(2.3) L=Wcos⁡γ

and

(2.4) $$D-\frac{\eta P}{v}=W\sin\gamma ,$$

which balance the hydrodynamic forces acting on the shark with gravity and thrust ηP/v. Here, *L* and *D* are the lift and drag defined in equations (2.1) and (2.2), *W* is the submerged weight, and *P* and *η* are respectively the mechanical power provided by the shark and the efficiency of translating this power into thrust. For a given angle of attack (the angle between the body and the swimming direction), the ratio of (2.4) and (2.3) determines the path angle, and the sum of their squares furnishes the swim speed. In a free glide, when no power is supplied by the shark, they yield;

(2.5) $$\gamma =\tan^{-1}\left(C_{D}/C_{L}\right)$$

and

(2.6) $$v=v_{ref}{\left(C_{L}^{2}+C_{D}^{2}\right)}^{-1/4},$$

in which

(2.7) $$v_{ref}=\sqrt{2W/\rho S},$$

is a combination of morphological parameters having the dimensions of speed. During active swimming, when *P* > 0, equations (2.3) and (2.4) (subject to equations (2.1) and (2.2)) have to be solved numerically. In both cases, the swim speed and the path angle can be converted into horizontal and vertical velocity components,

(2.8) $$v_{h}=v\cos\gamma \;and\;v_{v}=-v\sin\gamma .$$

Spanning the angle of attack between 0 and 16° (a shark can set its angle of attack by adjusting its body posture and its fins), the horizontal and vertical velocity components of a free-gliding model shark, obtained as the solution to equations (2.5)–(2.8) of equations (2.3) and (2.4), are plotted as a snail diagram [23] on figure 1c. Data were taken from wind tunnel experiments with a model shark [17,21,22]. The model predicts that our shark can minimize its sink rate by adjusting the angle of attack to 11° (point E on figure 1a–d), minimize its glide angle by lowering it to 9° (point G), maximize its horizontal speed component (aka the ‘penetration speed’) by lowering it further to 1° (point P) and maximize its vertical speed component (aka the ‘terminal speed’) by diving vertically at zero angle of attack (point T).

Adding power by active swimming raises the upper part of the snail diagram curve (figure 1d). The minimal power that allows the shark to swim at constant depth can be exploited at the same swim speed where the sink rate was minimal when gliding (point EB on the blue curve in figure 1d); it nullifies the vertical speed when swimming at an angle of attack of 11° (points E and B). With more power (shown in magenta), the shark can ascend or swim faster at a constant depth (point B, figure 1d).

If a shark is swimming into a current with upwelling, the ‘snail’ curve shifts upwards by the speed of the upwelling current, and to the left by the speed of the head-on current (figure 2). The stronger the upwelling current, the less power is needed to maintain depth, and when the speed of the upwelling exceeds the minimal sink rate of the shark (marked by the point E on the black curve on figure 1d) the shark can maintain depth with no power (in our model it would be 0.15*v*_ref_). At the same time, if the upwelling current is accompanied by a significant head-on component, which exceeds the respective horizontal speed of the shark (in our model shark it would be approximately 0.75*v*_ref_), the shark will need to use power to maintain position against the current (even though no power is needed to maintain depth).

Next we consider a pair of morphologically similar sharks, having the same *C_D_* versus *C_L_* relation as in figure 1b (and hence the same normalized snail diagram for a no-current situation as in figure 1c), but differing in their reference speed; one individual having a 1.5 times higher reference speed than the other. We do this to arbitrarily compare species of Carcharhinid shark that are similarly shaped but may differ in length and buoyancy (see below). Additional morphometric differences may further vary the reference speed. In a head-on upwelling current, their snail diagrams shift up and to the left by the (reduced) velocity of the current (figure 2). Although the current is the same for both sharks, the shark with the lower reference speed will experience a larger reduced current speed than the other (compare figure 2a,b). Assisted by the upwelling current, both sharks can now ascend at minimal power (blue curves in figure 2a,b), and hence, in principle, can reduce their routine metabolic rates compared to swimming in a location with no current. The shark with the higher reference speed can maintain position (horizontal and vertical) using 50% less power than if there were no current (cyan B in figure 2a). Its excess horizontal speed can be offset by its zigzagging left-right when swimming against the current. On the other hand, the shark with the lower reference speed must add additional power to maintain its horizontal position, more than required if there were no current (magenta B in figure 2b). In other words, the same location and conditions can be energetically beneficial for one individual (the one with higher reference speed, figure 2a), but detrimental for the other (figure 2b).

**Figure 2.**
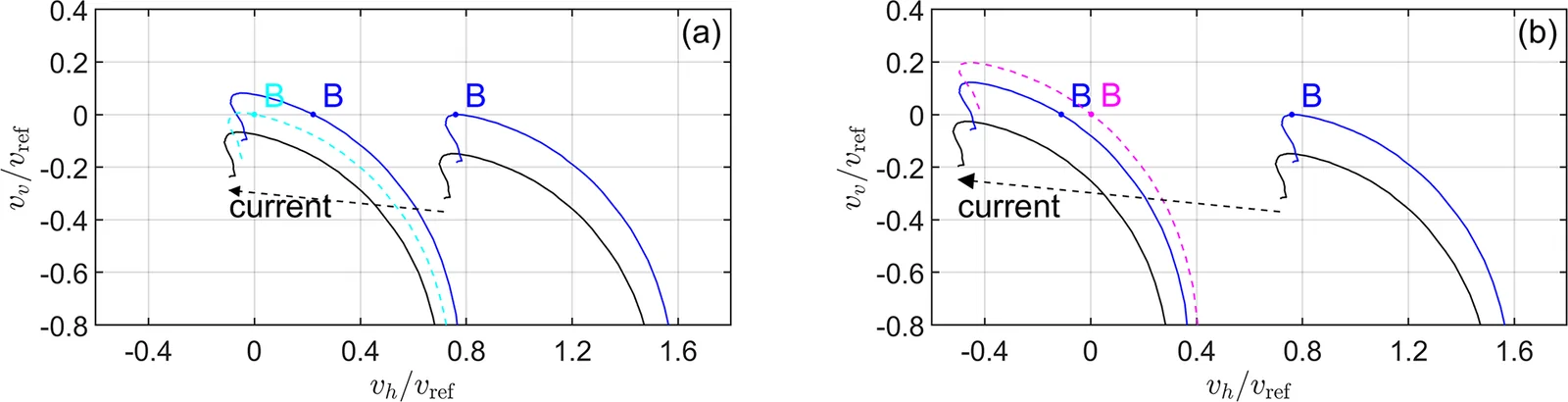
Effect of an upwelling head-on current on two similar sharks differing in their reference speeds (a,b), which is 1.5 times lower for the shark represented by the figure on the right (b). The pair of black and blue curves on the right of each plate are essentially the same as on figure 1d—the black curve represents an unpowered glide, the blue curve represents swimming at the minimal power that balances the respective animal at point B. A head-on upwelling current simply shifts these curves up and to the left by the velocity of the current. The current is the same for both plates, but because the reference speed of the shark on the right (b) is 1.5 times lower than that of the shark on the left (a), the reduced current speed appears 1.5 times larger. Assisted by the upwelling current, both sharks can now climb (ascend) at the minimal power, and hence, in principle, can reduce their routine metabolic rate below the minimal metabolic rate if the shark were not in a current. The shark on the left (a) can balance itself horizontally and vertically at half the power (cyan-coloured B) that would be required if there were no current. At the same time, the shark on the right (b) cannot keep its horizontal position without adding more power, at well above the minimal metabolic rate if there were no current (magenta-coloured B).

The reference speed is clearly a critical component of our model. Referring to equation (2.7), it can change with the cross-sectional area of the shark *S* and its submerged weight *W*. The latter can be estimated by the product;

(2.9) W≈βρgV,

in which *β* is the sinking factor (the relative buoyancy deficit), *g* is the acceleration of gravity and *V* is the volume of the body. In turn, the volume can be expressed as the product *V* = *k*_pc_*Sl*, where *l* is the fork length, *S* is the cross-sectional area of the shark (this is why it was used as the reference in equations (2.1) and (2.2)), and *k*_pc_ is the prismatic coefficient—the ratio between the volume of the body and the volume *Sl* of the minimal cylinder enclosing it. It is approximately 0.64 for our model shark (see [21] for details). Consequently,

(2.10) $$v_{ref}=\sqrt{2\beta k_{pc}gl},$$

changing mostly with size (the fork length) and the sinking factor (buoyancy). Two morphologically similar sharks having the same sinking factor, but one twice as long as the other, will have a 40% difference in their reference speed.

### (b) Reef sharks in a tidally flushed channel

The southern channel of Fakarava atoll, French Polynesia (16°18′ S 145°38′ W), connects to a large lagoon and is tidally flushed with current speeds estimated at approximately 0.5 m s^–1^ [6]. The channel contains over 500 sharks, the majority being grey reef sharks (*Carcharhinus amblyrhynchos*), but other species include blacktip reef (*Carcharhinus melanopterus*)*,* blacktip (*Carcharhinus limbatus*)*,* silvertip (*Carcharhinus albimarginatus*) and whitetip reef sharks (*Triaenodon obesus*) [24]. Grey reef sharks show very high residency in the channel throughout the year and are present day and night during both incoming and outgoing tides [24,25]. Sharks hunt reef fishes at night, but during the day grey reef sharks select key areas where there is upwelling during incoming tides to rest [6,24]. By selecting these upwelling regions, grey reef sharks may be able to reduce their metabolic costs by up to 15% [6]. We conducted dive surveys within the channel to determine the relative locations of other shark species that make up the overall aggregation. Briefly, divers started the survey close to the ocean drop-off during the incoming tide and drifted with the current through the centre of the channel while filming with a GoPro. Sharks swim against the incoming current (i.e. opposite the direction to the divers) and are not therefore attracted or disturbed by the divers. The surveys were terminated just before entering the lagoon (full details in [24]). The channel was divided into 22 sectors from South to North. Shark count surveys corresponding to 29 drifting dives from South to North during incoming tides allowed us to estimate relative shark abundance for each species across sectors. As the major upwelling sites are located in sectors S11–S12 and S17–S18, we restricted our survey to the section between sectors S09 and S18 [6]. Relative shark abundance was assessed using the maximum number of individuals of each species within a frame of the video (*MaxN*). For each dive, the highest number in each sector was retained. All dives were performed during incoming tides owing to low visibility during outgoing tides. Shark sizes were estimated by divers during the surveys (data in supplementary material). Our surveys show that silvertips, which are smaller juveniles (105 cm total length (TL); min = 86, max = 123), are mostly found in the centre of the channel, close to the seafloor in a region with minimal upwelling and within the boundary layer over the bottom, but do have some overlap with grey reef sharks (140 cm TL; min = 115, max = 165) (figure 3). Dive surveys and telemetry show that grey reef sharks primarily use the upwelling region during incoming tide (figure 3, [6]). Blacktip sharks, on the other hand (202 cm TL; min = 166, max = 238), are mostly seen in a very small section of the channel towards the centre and western region of the channel. Hence, these species, which differ in length and probably buoyancy, use different resting areas during daytime incoming tides. Of course, space use patterns could be due to other interactions, such as competition. However, no foraging occurs during the day, and there are still locations where species overlap (figure 3). It would appear unlikely that space is by itself a limiting resource, so we propose that these distribution patterns are driven by differences in the energy landscape.

**Figure 3.**
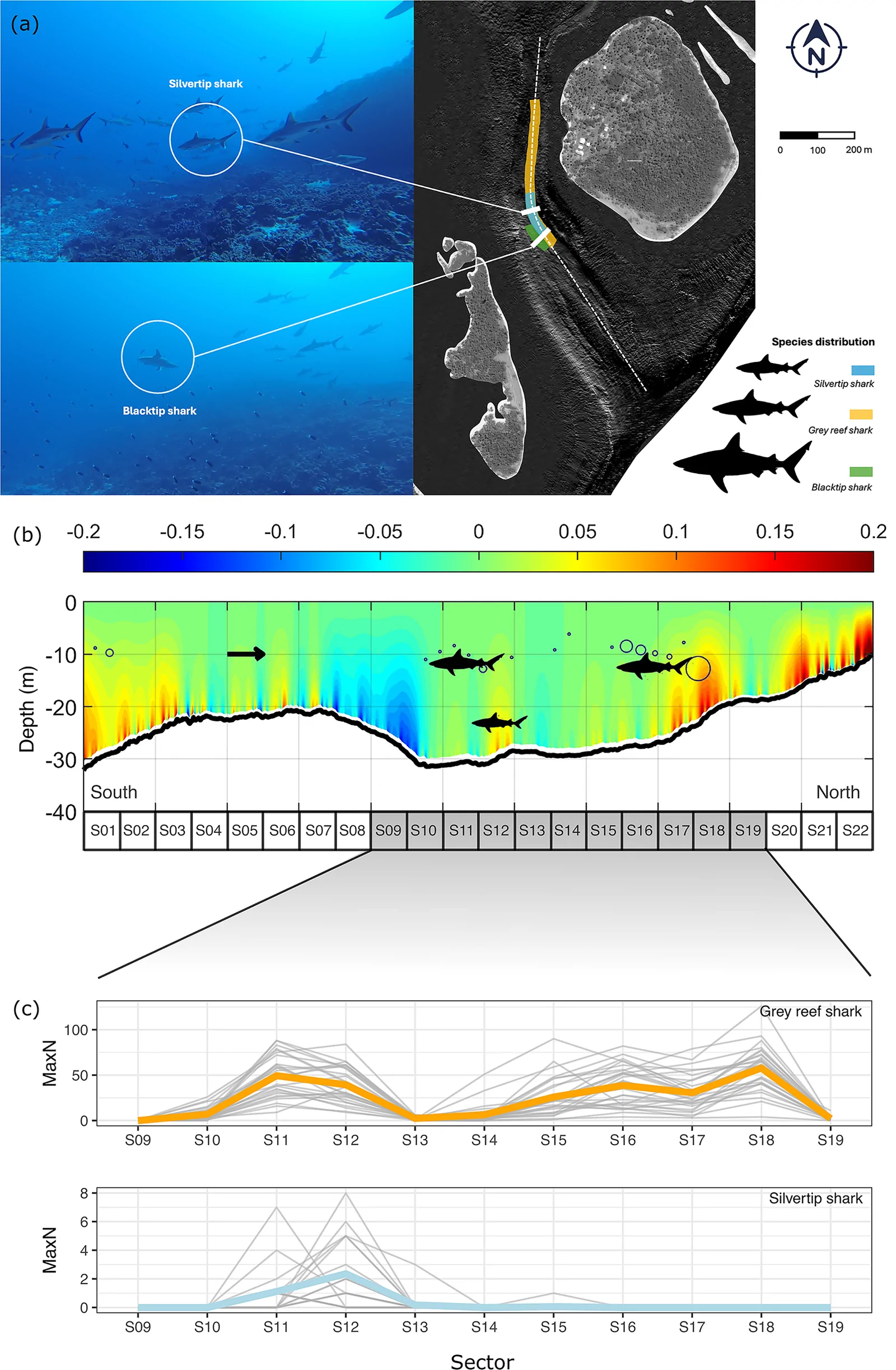
Distribution of three species of shark within the Fakarava Channel. (a) During the day, sharks select refuging sites with upwelling to save energy. While grey reef sharks are found at key sites throughout the channel, blacktips and silvertips are only found in a very narrow region of the channel. The relative size differences of the sharks are shown in the silhouettes. (b) The horizontal and vertical locations of grey reef (large silhouette) and silvertips (smaller silhouette) relative to predicted upwelling (red regions) and downwelling (blue) zones during the incoming tide (moving left to right). The circles show the swimming depth for grey reef sharks fitted with pressure-sensing acoustic transmitters (from [6]). (c) Each grey line represents the relative abundance (MaxN) for each species along the sectors. The thick colour lines are species averages across dives. These patterns may be driven by how the species interact with their energy landscape. The upwelling map in B is modified from [6].

## 3. Pelagic predator foraging on prey patches

A cornerstone of optimal foraging theory is predicting how long a predator should remain within a patch before abandoning it and searching for a new patch (marginal value theory). Traditional models predict that animals will forage so as to maximize their rate of prey capture [26]. More recent foraging theory considers additional constraints faced by foragers and predicts that animals will forage within a patch until a quitting harvest rate (*H*) is reached, defined by *H* = *C* + *P* + MOC [19]. Here, *C* is the energetic cost of foraging, *P* is the foraging cost of predation (i.e. risk of predation to the forager), and MOC is the missed opportunity costs (e.g. mating opportunities, better patches, etc.). Once the harvest rate drops below the quitting harvest rate, then the animal should abandon the patch as it is more beneficial to find a new one. Hence, a major determinant of quitting harvest rates is the energetic cost of foraging (*C*), which depends on the energy landscape [7]. Note that *C* is not an energy value but rather the harvest rate that makes it profitable to remain in the patch despite the energetic cost of foraging there.

Patches of prey fish can be found in the open ocean, but their distribution is spatially and temporally unpredictable. Foraging aggregations of predators may include sharks, teleosts, marine mammals and seabirds, and interactions between predators may range from competitive to mutualistic [14–16]. For our marine predators, *C* will be dependent on their reference speeds, current strength and dynamics, and potentially water temperature via its effect on metabolic rate (defined by the *Q*_10_, or how metabolic rate scales with a 10 °C change in body temperature; figure 4). For those species that are central place foragers (CPF), *C* will also depend on travel distance between the patch and the central place (e.g. a nest for a seabird) [3]. This is due to the CPF having to travel to the patch and back again, with energy costs dependent on the travel distance. There is a rich literature on CPF theory, with animals predicted to optimize their rate of prey intake by increasing residence time within patches as travel distances increase [27]. These models do not consider the energetic cost of travel, and distance from the central place is a component of the energy landscape [3]. Hence, greater travel distances to the patch could increase *H* (i.e. predators abandon the patch sooner) via an increase in *C*, which may counteract decisions based solely on maximizing the rate of prey capture (e.g. [3]).

**Figure 4.**
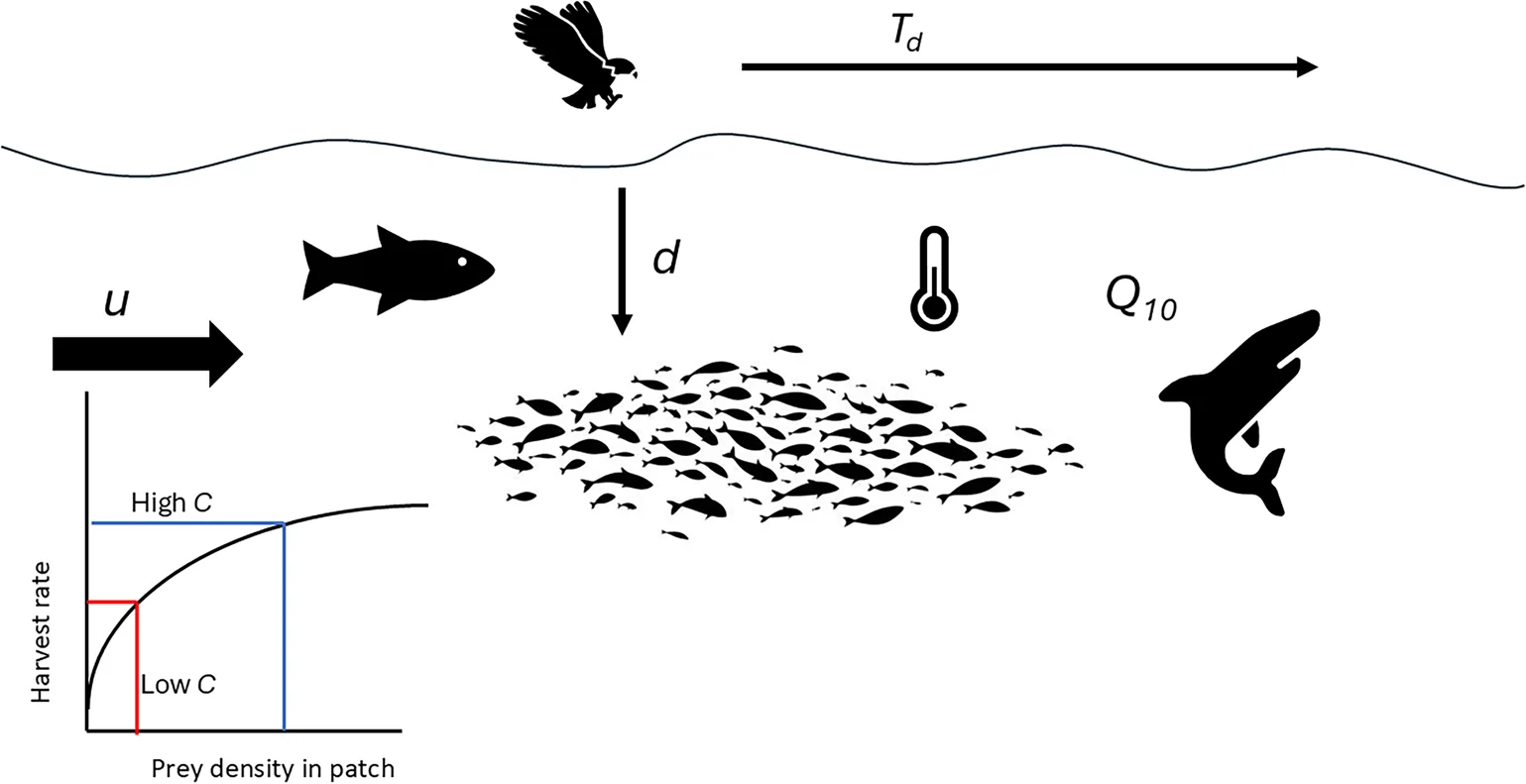
A multi-species predator aggregation foraging on offshore prey patches. Predators can include sharks, teleosts, marine mammals and birds. The energy landscape dictates the energetic cost of foraging in the patch and depends on diving depth (*d*), current speed (*u*), travel distance to the central place (*T_d_*) and water temperature. These will differ for each species based on factors, such as morphometrics, buoyancy, length and the *Q*_10_. The inset shows the relationship between a predator's harvest rate in a depletable patch and the density of prey in the patch. If the energetic cost of foraging *C* is high, then the predator will abandon the patch at a higher harvest rate and with a higher density of prey remaining in the patch.

For a multi-species predator aggregation, the species *H* and residence times in patches will more specifically depend on their size, buoyancy, morphometrics, behaviour (CPF versus roaming), as well as thermal physiology (endothermic versus ectothermic, *Q*_10_, etc.). Subsequent differences in residence times may have consequences for other predators in the aggregation, both positive (loss of competitors) [15] and negative (loss of a mutualistic interaction) [16].

## 4. Discussion

We describe two potential scenarios where the energy landscape may influence multi-species predator aggregations in marine ecosystems. These include refuging aggregations as well as foraging on shared prey resources. Within both scenarios, it is important to consider the spatial scale over which aggregations occur and their hierarchical nature. Large aggregations may occur over broad areas, but nested within these are small-scale and higher-density aggregations [28]. The drivers of this spatial scale and hierarchical structure may vary (e.g. prey patches and refuging habitats) but will have important implications for how species interact with their respective energy landscapes.

### (a) Refuging in predators that never stop swimming

Negatively buoyant sharks that never stop swimming may reduce their metabolic costs by remaining within upwelling currents, although this will depend on the current strength [6]. Our model shows how the energy landscape, in this case defined by upwelling currents, may cause habitat separation between sharks of different length and/or buoyancy. The reference speed, which dictates energy savings or costs in an upwelling, is a function of buoyancy and shark length. Therefore, even with all other morphometrics being identical, differences in shark length can turn an upwelling current from energetically beneficial to detrimental. Upwelling may still be beneficial to all species (if the difference in reference speed is not large), but the extent of this benefit will vary. Of course, there may be species within a multi-species aggregation that are not morphologically similar and will vary in factors such as fin span, lift surface areas (e.g. pectoral fins) and body diameter, all of which influence swimming performance [17]. Furthermore, there can be ontogenetic changes with negative allometry of caudal fin area in some species [29], while buoyancy may increase as individual sharks get older [17]. These could also lead to significant differences between sharks of varying sizes and ages, even when they are of the same species. Overall, we would predict that sharks are likely to use upwelling sites with other individuals/species that are of similar length, buoyancy and morphometrics.

These differences may then lead to spatial heterogeneity of species distributions within multi-species aggregations, as individuals select the microhabitats that are most energetically profitable for them. Patterns of space use can then determine opportunities to associate with other individuals and potentially drive assortativity. Individual physiology has been linked to patterns of assortativity within fish shoals (e.g. individual aerobic scope), but here, we show how morphology and the energy landscape could also play a role, even between different species [10,18]. We show evidence for this from Fakarava Channel, where grey reef, silvertip and blacktip sharks all use different regions of the channel (with some overlap). Silvertip sharks in the channel are juveniles and therefore small, which may explain why they avoid the major upwelling zone and only remain close to the bottom (where the current is weaker). By having a lower reference speed, the major upwelling zones may not be energetically beneficial, unlike for the larger grey reef sharks, which may also be more buoyant [17]. Swimming kinematic and morphometric data from silvertips in the channel are required to test these ideas. During the outgoing tides, grey reef sharks switch their space use and swimming depth as upwelling zones now become downwelling [6,25]. Turbulence is also generated during the outgoing tide, which impacts the behaviour of the sharks (they disperse and swim in shallower water) and may even limit foraging [6,25]. The energy landscape during the outgoing tide may further reduce the associations between different species.

The energy landscape may influence patterns of associations between species but may also drive the outcome of interactions, such as competition or predation. For example, when sharks are foraging (e.g. in the Fakarava Channel at night), their foraging efficiency relative to competing species will be dependent on their swimming abilities and metabolic costs. Somewhat analogously, wind dynamics contribute to competitive outcomes between species of soaring birds dependent on their wing shape [30]. Within the channels of French Polynesia, great hammerhead sharks (*Sphyrna mokarran*) are known predators of grey reef sharks [31]. Owing to their greater length and large sinking factor [32], hammerheads should have higher reference speeds and may be better equipped to benefit from strong tidal currents. Current speeds vary between channels and atolls, which may partially explain why great hammerheads tend to be more abundant in high current channels [33]. More accurate measures of buoyancy are needed so that interspecific comparisons of reference speeds can be made.

### (b) Pelagic predators foraging on prey patches

A great deal of theoretical work has gone into predicting patch residence times of foragers and how these relate to harvest rates [19]. Modern optimal foraging theory incorporates the energetic cost of foraging in a patch and the risk of predation to the forager, but considerably more research has gone into the effects of predation risk [19,34]. However, the energetic cost of foraging will be directly related to the energy landscape, and we identify how this might apply to predators foraging on fish schools. Pelagic predators, including tuna, billfish, sharks, marine mammals and birds, may forage on bait-fish in pelagic waters [14,16]. While upwelling will not be an issue, current speeds will vary and may still interact with predator length and/or buoyancy. The depth of prey patches will play a role, as the energy landscape of diving animals will depend on the travel distance (especially for CPF) and metabolic costs of diving, and potentially the scaling of metabolic rate with temperature [3,4,35]. Although depth may initially appear to apply only to air-breathing species, large fishes (teleosts and sharks) must also return to surface waters to access warmer waters to maintain cardiac (and digestive) function and growth rates, as well as a metabolic advantage over their prey (as they will have a higher body temperature than cold prey in deeper water) [4]. The costs to exploit prey patches at certain depths will therefore vary between species [4,35]. Therefore, the energetic cost of foraging may be species-specific, which will also alter their quitting harvest rates.

Quantitative tools for analysing animal movements, such as the first passage time (FPT) [36], can measure residence times of marine animals in patches. These have further been modified to quantify patch use in three dimensions (3D FPT or volume-restricted searching) and to detect hierarchical searching, where smaller patches are nested within larger ones [37]. These are important as multi-predator aggregations may similarly form over multiple spatial scales to match those of the prey patches on which they are foraging. Patch leaving decisions will also depend on the abundance and proximity of other patches (‘missed opportunities’ in the foraging model) [38], and these may also interact with the energy landscape in different ways (e.g. travel distances for CPFs, current strength, etc.). Extensive theoretical work on marginal value theory has been developed but should consider the interaction with the energy landscape, and how this may vary for different predators foraging on the same patches.

## 5. Conclusion

Using a modelling and conceptual framework, we highlight two mechanisms by which the energy landscape may interact with multi-species predator aggregations. The importance of the energy landscape in shaping animal movements and habitat selection is now well established [1], but future studies should also consider how it may interact with and influence multi-species aggregations during refuging and foraging. These interactions may drive patterns of assortments within aggregations and/or changes in species composition while refuging and foraging. Future compositions should incorporate detailed measures of morphometrics and buoyancy, with biologging to measure field metabolic rates. Biologging and motion-sensing sensors (e.g. accelerometers) enable researchers to measure field metabolic rates and how these vary by habitat and with behaviours [3,4,39]. These quantitative measures of habitat-specific power costs will allow these predictions to be tested.

## Supplementary Material



## Data Availability

Data are included in the supplementary material [40].

## Theme

One contribution of 14 to a theme issue ‘Multi-predator feeding aggregations in pelagic waters’.
